# Examination of the Diameter of the Rectum and the Thickness of the Anterior Wall of the Rectum by Ultrasound in Children With Chronic Constipation and Abdominal Pain in the Age Range of 2–18 Years

**DOI:** 10.1002/jgh3.70202

**Published:** 2025-06-29

**Authors:** Mitra Azra Aldaghi, Nafiseh Shourideh Yazdi, Mohammad Esmail Ahmad Abadi, Rahil Mahmoudi, Hadi Lotfi

**Affiliations:** ^1^ Department of Pediatrics Faculty of Medicine, Sabzevar University of Medical Sciences Sabzevar Iran; ^2^ Iranian Research Center on Healthy Aging Sabzevar University of Medical Sciences Sabzevar Iran; ^3^ Leishmaniasis Research Center Sabzevar University of Medical Sciences Sabzevar Iran

**Keywords:** constipation, rectal diameter, rectal thickness, ultrasonography

## Abstract

Abdominal pain is one of the most common complaints to the pediatric clinic and can be challenging for the physician. Among children who present with abdominal pain, constipation is one of the main causes of abdominal pain, and its incidence in childhood is estimated at 1%–30%.

**Materials and Methods:**

Hundrerd children with chronic constipation, defined according to the Rome IV constipation criteria, who were referred to Sabzevar Children's Clinic, were included in the study. Hundred people in the control group were selected from children without constipation who underwent abdominal ultrasound due to abdominal pain. Consent was obtained from the children's parents, and the child's consent was also obtained for ultrasound. Rectal diameter and rectal anterior wall thickness were measured by an experienced radiologist.

**Results:**

The results determined that the diameter of the rectum measured by ultrasound in children with constipation is greater than in the control group. Also, the thickness of the rectum was lower in children with constipation compared to the control group. It was found that there was no significant relationship between gender and constipation in children. There was a significant relationship between constipation in children and their body mass index (BMI) and age. The results of the study showed that there was no significant relationship between history of urinary tract infection and constipation and socioeconomic status in children. There was a significant relationship between the diameter of the rectum and the duration of constipation.

**Discussion:**

Ultrasound can be useful in diagnosing children with constipation in whom it is difficult to take a history and physical examination.

## 1 Introduction

Abdominal pain is one of the most common complaints in children's clinics and can be challenging for doctors. Among children who present with abdominal pain, constipation is one of the main causes of abdominal pain, and its occurrence in childhood is estimated at 1%–30% [1, 2]. Constipation is associated with significant complications such as digestive system dysfunction, urinary tract infection, enema treatment for defecation, irritable bowel syndrome and many other complications [3]. Constipation has a significant impact on a person's lifestyle. Constipated patients need medical care and spend more money. In a study, patients with constipation needed two times more medical care and four times more expenses than the healthy control group [4]. To diagnose constipation in children, history taking and physical examination may be unreliable; part of the physical examination included a digital rectal examination (rectal bag) which, due to the discomfort and invasive nature of this examination, in practice, 85% of doctors do not perform rectal examination, while 10% or less than 5% of patients who have abdominal pain actually perform rectal examination [5]. Due to the uncertainty of taking a history and physical examination, doctors usually perform an abdominal radiograph to support the diagnosis of constipation and rule out other injuries. Abdominal radiography has been found to be unreliable in diagnosing constipation, with sensitivity ranging from 60% to 80% and specificity ranging from 35% to 90%, while exposing the child to potentially harmful ionizing radiation [6]. Another suggested method for diagnosing constipation is using ultrasound. From the point of view of care, ultrasound is used in many diagnoses in the emergency department and children's clinics, and therefore patients are more satisfied with it [7]. Measuring the diameter of the rectum using ultrasound has been studied in children's surgery and urology patients.

## 2 Materials and Methods

Children with chronic constipation defined according to the Rome IV constipation criteria and referred to Sabzevar Children's Clinic were included in the study. Constipation was diagnosed according to the Rome IV criteria, which require the presence of at least one of the following symptoms in children:

1. **Infrequent defecation** (≤ 2 bowel movements per week).
2. **Fecal incontinence** (≥ 1 episode per week).
3. **History of retentive posturing or fecal retention** (observed or reported).
4. **Painful or difficult defecation** (dyschezia).
5. **Passage of large‐diameter stools** capable of obstructing the toilet.
6. **Palpable fecal mass** (hard stools) on digital rectal examination.

### 2.1 Pediatric Patients Aged 2–18 Years

Given that rectal diameter and wall thickness are well‐defined sonographic parameters, we aimed to investigate constipation as a primary etiology of abdominal pain. For this comparative study, we enrolled patients who presented to our clinic with a clinical diagnosis of constipation, excluding those with a prior history of constipation to minimize confounding factors.

The control group is selected from children without constipation who underwent abdominal ultrasound due to abdominal pain. A consent letter is obtained from the parents of the children, and the child's consent is also taken to perform the ultrasound. Children with known causes of constipation (such as Hirschsprung's disease, anorectal/pelvic abnormalities), muscle disorders, or previous anorectal surgery were excluded. No rectal examination was performed before the ultrasound. The measurement of the diameter of the rectum and the thickness of the anterior wall of the rectum is done by an experienced radiologist.

## 3 Result

### 3.1 Investigating the Relationship Between Age and Socioeconomic Status in Children With Constipation and Children Without Constipation

The comparison between children living in urban and rural areas was based on differences in their dietary habits and socioeconomic status. The average age in 100 patients with constipation was (3.03 ± 7.65 years) and in the group of control and nonconstipated children was (2.49 ± 7.75 years). There was a significant relationship between the age of the subjects and constipation (*p* = 0.1 < 0.05). The average diameter of the rectum in patients with constipation who lived in the city was (21.53 ± 6.13 mm). The average diameter of the rectum in affected patients who lived in the village was 23.33 ± 7.82 mm. There was no significant relationship between the diameter of the rectum in patients with socioeconomic status (*p* = 0.07 > 0.05). The average thickness of the rectum was 1.66 ± 0.34 mm in affected patients who lived in the city and 1.72 ± 0.55 mm in affected patients who lived in the village. There was no significant relationship between rectal thickness in affected patients and socioeconomic status (*p* = 0.3 > 0.05).

### 3.2 Determination of the Diameter of the Rectum in the Studied Population

The average diameter of the rectum in 100 patients with constipation was (24.81 ± 7.41 mm; minimum and maximum 50–13 mm, respectively). Also, in the control group, the average diameter of the rectum was (19.45 ± 4.76 mm; 32–11 mm). There was a significant relationship between the diameter of the rectum and constipation (*p* = 0.001 < 0.05).

### 3.3 Determination of Rectal Thickness in the Studied Population

The average thickness of the rectum in 100 patients with constipation was (1.68 ± 0.47 mm; 1–4 mm). Also, in the control group, the average thickness of the rectum was (1.69 ± 0.36 mm; 1–3 mm). There was no significant relationship between rectal thickness and constipation disease (*p* = 0.94 > 0.05) (Table 1).

**TABLE 1:** Rectal thickness and diameter in the studied population.

	Rectal thickness (mili meter)	The diameter of the rectum (mili meter)	*p*
Constipation group	1.68 ± 0.47	24.81 ± 7.41	0.94
Nonconstipated group	1.69 ± 0.36	19.45 ± 4.76	0.86

### 3.4 Determining the Duration of Constipation in the Group of People With Constipation

In 100 patients with constipation, the average duration of constipation was (22.33 ± 16.26 months; minimum 1–maximum 72 months).

### 3.5 Investigating the Relationship Between Gender and Constipation in Children With Constipation and Children Without Constipation

Out of 100 patients with constipation, 39 were boys and 61 were girls. There were also 63 girls and 37 boys in the control group. There was no significant relationship between gender and constipation in the studied subjects (*p* = 0.8 > 0.05).

### 3.6 Investigating the Relationship Between Socioeconomic Status and Constipation in Children With and Without Constipation

Out of 100 patients with constipation, 68 people lived in the city and 32 people lived in the village. Also, in the control group, 66 people lived in the city and 34 people lived in the village. There was no significant relationship between socioeconomic status and constipation in the studied subjects (*p* = 0.8 > 0.05).

### 3.7 Investigating the Relationship Between Rectal Diameter and Rectal Thickness With Constipation in Children With and Without Constipation

In 100 patients with constipation, the average diameter of the rectum was (24.81 ± 7.41; 50–13 mm). In the control group, the average diameter of the rectum was (19.45 ± 4.76; 32–11 mm). The diameter of the rectum was greater in people with constipation than in the control group. There was a significant relationship between the diameter of the rectum and constipation in the studied subjects (*p* = 0.001 > 0.05). The average thickness of the rectum in 100 patients with constipation was (1.68 ± 0.47; 1–4 mm). In the control group, the average rectal thickness was 1.29 ± 0.36 (0.7–3) mm. There was no significant relationship between rectal thickness and constipation in the studied subjects (*p* = 0.94 > 0.05).

### 3.8 Relationship Between Diameter andThickness of Rectum With Gender inChildren With Constipation

The average diameter of the rectum in 61 female patients with constipation was (21.49 ± 6.67 mm) and in 39 male patients with constipation it was (23.17 ± 6.85 mm). There was no significant relationship between the diameter of the rectum and gender in patients with constipation (*p* = 0.08 > 0.05). The average thickness of the rectum in 61 female patients with constipation was 1.71 ± 0.44 mm and in 39 male patients with constipation it was 1.64 ± 0.37 mm. There was no significant relationship between rectal thickness and gender in patients with constipation (*p* = 0.27 > 0.05).

### 3.9 Correlation Between Diameter and Thickness of Rectum With History of Urinary Tract Infection

The average diameter of the rectum was (22.22 ± 5.22 mm) in patients who had a history of UTI and 22.10 ± 7.16 mm in patients who did not have a history of UTI. There was no significant relationship between the diameter of the rectum in patients with constipation and previous history of urinary tract infection (*p* = 0.9 > 0.05). The average rectal thickness was 1.75 ± 0.3 mm in patients who had a history of UTI and 1.64 ± 0.44 mm in patients who did not have a history of UTI. There was no significant relationship between rectal thickness in patients with constipation and previous history of urinary tract infection (*p* = 0.2 > 0.05).

### 3.10 The Relationship Between the Diameter and Thickness of the Rectum With the Duration of Constipation

There was a significant relationship between the diameter of the rectum and the duration of constipation in the studied patients (*r* = 0.48, *p* = 0.001 > 0.05). As the duration of constipation increased, the diameter of the rectum was larger. There was no significant relationship between rectal thickness and duration of constipation in the studied patients (*r* = 0.01, *p* = 0.8 > 0.05).

### 3.11 The Relationship Between the Diameter and Thickness of the Rectum With Age

There was no significant relationship between the diameter of the rectum and age in the studied patients (*r* = 0.13, *p* = 0.05 > 0.05). There was no significant relationship between rectal thickness and age in the studied patients (*r* = 0.13, *p* = 0.06 > 0.05).

### 3.12 Relationship Between Rectal Diameter and Thickness With Body Mass Index (BMI)

There was a significant relationship between the diameter of the rectum and BMI in the studied patients with constipation (*r* = 0.08, *p* = 0.03 < 0.05). There was no significant relationship between rectal thickness and BMI in the studied patients with constipation (*r* = 0.15, *p* = 0.2 > 0.05) (Table 2). The results of our study showed that the biggest difference in both the thickness and the diameter of the rectum was related to the third and fourth BMI groups.

**TABLE 2:** BMI in the studied children.

	Less than 18/5 (kg/m^2^)	18/5–24/9 (kg/m^2^)	25–29/9 (kg/m^2^)	More than 30 (kg/m^2^)
Number	65	101	27	7

## 4 Discusion

Constipation is one of the most common reasons for outpatient visits to pediatric care or surgery. The most common cause of constipation in children is functional constipation, which leads to the enlargement of the rectum due to retention of stool. The Rome IV criteria are currently used to evaluate and define constipation. Patients are evaluated according to the history and physical examination based on a series of acquired scores. Evaluation of rectal dilation due to constipation is not possible with these criteria. The present study showed that it is possible to diagnose constipation in children between the ages of 2 and 18 by determining the diameter of the rectum and the thickness of the anterior wall of the rectum by ultrasonography. The results of our study determined that the diameter of the rectum in children with constipation is greater than in the control group. In other words, the rectum was thicker in patients with constipation. In Momeni et al.'s study in 2019, with the title of ultrasound in determining the diameter of the rectum and the thickness of the rectal wall in children with and without constipation, they concluded that the diameter of the rectum in children with constipation was greater than in children without constipation [8]. The results of our study confirmed that the diameter of the rectum was greater in patients with constipation than children without constipation. In Momeni et al.'s study in 2019, it was found that there was no significant relationship between gender and constipation [8]. The results of our study also revealed that there was no significant relationship between gender and constipation in children.

In Misra et al.'s study titled Chronic Constipation in Overweight Children, they concluded that there seems to be a relationship between severe chronic constipation and overweight. Children with constipation were more overweight compared to the control group [9]. The results of our study confirmed this.

In the study of Kaveh Menesh et al., comparing BMI in children with functional constipation and controls, they concluded that there was a significant relationship between the presence of constipation and the duration of constipation with obesity [10]. The results of our study also showed that there is a significant relationship between constipation in children and their weight gain and BMI. In other words, weight gain may cause constipation.

The results of our study showed that there was a significant relationship between age and constipation in children. With increasing age, the probability of constipation in children was higher. In Sarvari et al.'s study titled the relationship between chronic constipation and urinary tract infection in children: a clinical case–control study, they concluded that despite the absence of significant urinary tract infection between children with constipation and children without constipation, constipation should still be considered as a should be considered as a predisposing risk factor for the occurrence of urinary tract infection [11]. The results of our study also showed that there was no significant relationship between the history of urinary tract infection and constipation in children.

In the study of Sampaio et al. entitled constipation and lower urinary tract dysfunction in children and adolescents, they concluded that there was no significant relationship between constipation and infection and urinary tract dysfunction [12]. The results of this study confirmed the results of our study.

In our study, it was found that there is no significant relationship between constipation in children and socioeconomic status. In the study by Berg et al. entitled “Epidemiology of Constipation in Children,” it was found that socioeconomic status had no significant relationship with constipation in children [7].

In our study, there was a significant relationship between the diameter of the rectum and the duration of constipation. As the duration of constipation increased, the diameter of the rectum increased. In the study of Imanzadeh et al. entitled Measuring the diameter of the rectal ampulla with abdominal ultrasound as a less invasive alternative to digital rectal examination in children with functional constipation, they concluded that the rectal diameter increased with the increase in the period of constipation [13].

In the study of Yovanita et al. titled obesity and functional constipation in children, they concluded that there is a significant relationship between obesity and increased BMI with constipation in children [14]. In our study, there was a significant relationship between diameter and BMI of the study subjects. With the increase in BMI and the weight of the studied subjects, the diameter of the rectum increased and there was an increase in the probability of constipation.

## 5 Conclusion

Ultrasound can be useful in constipated children in whom history and physical examination are difficult. Ultrasound can also be used to detect fecal retention in children with constipation.

## Conflicts of Interest

The authors declare no conflicts of interest.
